# Exposure to the Riot Control Agent CS and Potential Health Effects: A Systematic Review of the Evidence

**DOI:** 10.3390/ijerph120201397

**Published:** 2015-01-27

**Authors:** Yiannis Dimitroglou, George Rachiotis, Christos Hadjichristodoulou

**Affiliations:** Department of Hygiene and Epidemiology, Medical Faculty, University of Thessaly, 22 Papakyriazi Str., Larissa 41222, Greece; E-Mails: dimiyann@hotmail.com (Y.D.); xhatzi@med.uth.gr (C.H.)

**Keywords:** CS, riot control, exposure, health effects, systematic review

## Abstract

*o*-Chlorobenzylidene malononitrile (CS) is one of the most extensively used riot control agents. Our aim was to conduct a systematic review of the potential health effects related to CS exposure. We searched for papers in English between 1991 and 2014. Thirty five (35) studies (25 case reports, seven descriptive studies and three analytical studies) were included in the review. In the twenty five case reports/series 90 cases of exposure to CS and their clinical effects are presented. Their mean age was 25.7 years and 62.0% were males. In addition, 61% of the cases described dermal, 40% respiratory, 57% ocular clinical effects. Life threatening situations as well as long-term health effects were found and were related with exposure to confined/enclosed space. Descriptive and analytical studies have shown attack rates ranging from 12% to 40%. Subjects who were sprayed by the police more often needed special treatment and reported adverse health effects. Apart from transient clinical effects, CS could have lasting and serious effects on human health. Better surveillance of the subjects exposed to CS and completion of cohort studies among exposed populations will illuminate the spectrum of the health effects of exposure to CS.

## 1. Introduction

Riot control agents include a variety of chemical substances; however, *o*-chlorobenzylidene malononitrile (CS) is the most commonly available riot control agent. It has been suggested that use of CS is characterized by rapid onset of effects, brief duration of effects and minimal side-effects [1,2,3,4,5]. However, there are available data which refer to long-term effects and even life-threatening consequences [6,7,8]. The mechanism of action of CS is not yet fully clarified. It has been proposed that CS is an alkylating agent that reacts with glutathione, SH-containing enzymes, proteins and nucleic acids. Data on CS mutagenicity are controversial [9,10,11]. Moreover, the solvent of CS (MIBK) is believed to be hazardous [4,12], but it’s health effects have not been investigated well [4,13]. To our knowledge there is no systematic review of the health effects of exposure to CS.

## 2. Experimental Section

### Methods

**Figure 1:**
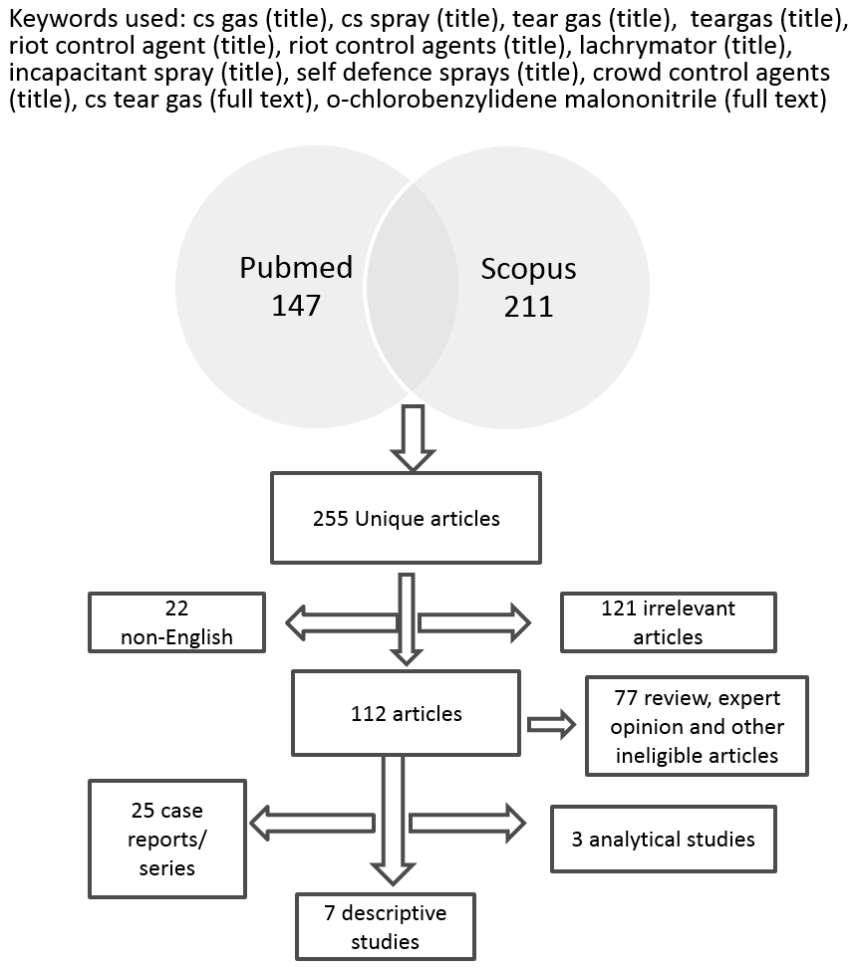
Process of the systematic review.

We searched in PubMed and Scopus for articles in English language published between 1991 and 2014 about the health effects of exposure to CS. The following keywords were used: cs gas (title), cs spray (title), tear gas (title), teargas (title), riot control agent (title), riot control agents (title), lachrymator (title), incapacitant spray (title), self defence sprays (title), crowd control agents (title), cs tear gas (full text), *o*-chlorobenzylidene malononitrile (full text). In total, 255 articles were found, especially, 147 in PubMed, 211 in Scopus and 103 in both databases. From these 255 articles 121 described topics irrelevant to the health effects after exposure to CS and 22 were written in a language other than English. From the remaining 112 papers, 35 were considered eligible for our review (25 case reports/series, seven descriptive studies and three analytical studies. The selection process is summarized in Figure 1.

Symptoms have been classified in four categories; dermal, respiratory, ocular and other and attempt has been made to record the latency period of each category of symptoms. We also attempted to present the case reports according to the following parameters: Type of exposure (occupational or non-occupational), specific exposure conditions (agent on air, CS spray, direct contact CS canister, secondary exposure), exposure duration (CS spray), exposure distance (CS spray), part of the body exposed (CS spray) or affected (dermal symptoms), latency period and type of symptoms, duration of symptoms, hospital admission, way of treatment and long-term consequences. Two independent reviewers extracted the relative data from each paper on to a standard record sheet. All disagreements were settled through discussion with a third reviewer.

## 3. Results

Thirty five (35) studies were included in the systematic review. Among them 25 were case reports or case series, seven (7) were descriptive studies and three (3) were analytical studies (Table 1 and Table 2).

**Table 1:** Case reports/series included in the study.

Year	Author	n	Country	Reference
2012	Bhargava *et al.*	1	UK	[14]
2011	Shamphu *et al.*	1	UK	[15]
2011	Wu *et al.*	1	UK	[16]
2010	Kain *et al.*	1	UK	[17]
2009	Agrawal *et al.*	1	UK	[5]
2009	Karaman *et al.*	1	TURKEY	[18]
2006	Hardwicke *et al.*	1	UK	[19]
2005	Horton *et al.*	7	USA	[20]
2005	Watson *et al.*	7	UK	[21]
2005	Morrone *et al.*	1	ITALY	[22]
2004	Davey *et al.*	3	UK	[23]
2003	Solomon *et al.*	7	ISRAEL	[24]
2003	Horton *et al.*	5	USA	[25]
2001	Southward *et al.*	1	UK	[13]
2001	Varma *et al.*	1	UK	[26]
2000	Barlow	1	UK	[27]
2000	Hill *et al.*	1	USA	[28]
1999	Sommer *et al.*	1	UK	[29]
1998	Breakell *et al.*	23	UK	[30]
1997	Kiel *et al.*	6	UK	[31]
1996	Roth *et al.*	1	USA	[32]
1993	Bhattacharya *et al.*	2	UK	[33]
1993	Parneix-Spake *et al.*	11	FRANCE	[34]
1992	Hu *et al.*	1	USA	[35]
1991	Ro *et al.*	2	SOUTH KOREA	[36]

**Table 2:** Descriptive or analytical studies included.

Year	Author	No of Cases	Study Type	Country	Reference
2014	Hout *et al.*	5298	Analytical	USA	[37]
2014	Hout *et al.*	6723	Analytical	USA	[38]
2007	Hankin *et al.*	21	Descriptive	UK	[39]
2004	Euripidou *et al.*	152	Descriptive	UK	[40]
2003	Nathan *et al.*	30	Descriptive	UK	[41]
2003	Karagama *et al.*	34	Analytical	UK	[42]
2002	Thomas *et al.*	38	Descriptive	USA	[43]
1998	Wheeler *et al.*	597	Descriptive	UK	[7]
1996	Anderson *et al.*	184	Descriptive	HONG KONG	[44]
1995	Zekri *et al.*	96	Descriptive	HONG KONG	[45]

### 3.1. Case Reports/Case Series

In the twenty five case reports/series 90 cases of exposure to CS and their clinical effects are presented. The mean age was 25.7 years and 62.0% were males (38.0% females).

### 3.2. Conditions of Exposure to CS

**Figure 2:**
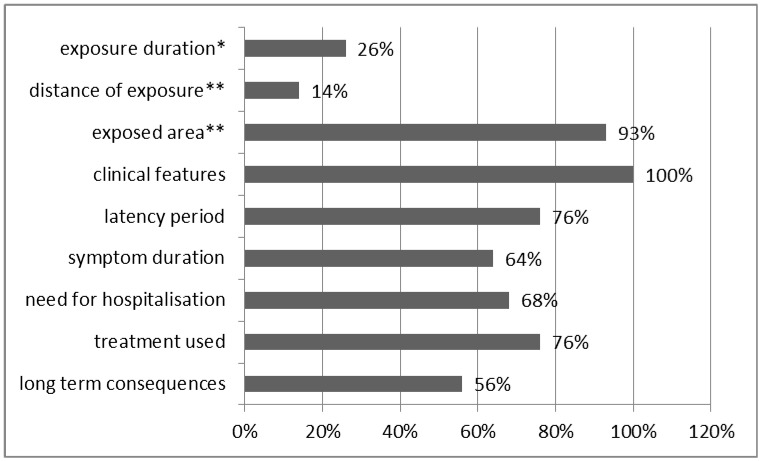
How often case reports report basic elements of exposure and clinical effects related to CS.

Regarding exposure status 22.7% of the cases referred to occupational and 77.3% to environmental exposure. Two studies reported secondary occupational exposure of five health care workers (emergency department personnel) [20,25] and another three studies reported secondary exposure of an anaesthetist following surgical procedure [23,27,33]. Notably, 11 cases were exposed during altercations with the police and six of them were exposed during riots. Six studies reported direct exposure (sprayed by the police) during altercations with the police against the subject’s face. Indirect exposures accounted for 52% of all exposures (35% agent on air after cartridge or spray use, and 17% secondary exposure). Direct contact with CS accounted for 41% of all exposures (30% CS spray; 8% ingestion, 3% direct contact with the substance) In addition, 2.5% of all exposures referred to direct canister hits. In 4.5% the type of exposure was not stated in the article. Only 26% of the case reports provided information on duration of exposure, which varied between 10–30 s [32,33] and 10–15 min [26,35]. We have limited available data about the distance between the spray and the exposed body surface. Exposures from a distance of 12 inches or 30 cm have been reported [5]. On the other hand 92% of the case reports described the part of the body affected and three case reports reported the total body surface percentage affected [5,19,34]. In particular Parneix-Spake *et al.* presented 11 cases with 2–13% surface affected and an average of 8% [34], Hardwicke *et al.* presented a case with 7% of the body surface affected with partial thickness burns [19], while Agrawal *et al.* presented a case of a burn affecting 4% of body surface [5]. From the 12 cases reports that included available data, all reported use of spray directed against the subject’s face. [5,13,15,16,17,19,21,25,26,28,31,33,34]. In five of them the neck was sprayed [5,19,21,28,34], in five the hands or arms [16,17,25,28,34], in three the chest [16,19,28] and the waist or the thigh [16,21]. Finally, a considerable percentage of case reports didn’t include information on various clinical outcomes after exposure to CS. In particular, 24% of the case reports didn’t describe the latency period between exposure to CS and related clinical effects. Further, 36% of the case reports didn’t provide data on the duration of symptoms (Figure 2).

### 3.3. Clinical Effects of Exposure to CS

The duration of symptoms varies between different types of symptoms. In most patients with ocular symptoms such as lacrimation or burning sensation of the eyes recession of the symptoms was observed within minutes or a few hours [21,23,24,25,27,31]. However, a case of conjunctivitis which lasted for 2 days was presented by Bhattacharya *et al.* [33]. Brief duration of symptoms has been reported regarding respiratory irritation [24,25], while chest tightness may last for one day [30]. However, complications may last for months and up to two years in case of Reactive Airways Dysfunction Syndrome (RADS) [28,32,35]. Erythema may last for a few days to one week [21,36] while vesicular eruptions, blistering rash or diffuse swelling usually subsides within days [13,35] or up to 4 weeks [5,21,26]. In the case of AGEP presented by Wu *et al.* the symptoms persisted for more than two weeks [16]. In addition, in the case presented by Hill *et al.* the dermatitis lasted for several months [28]. In total, among the 90 cases presented, 61% describe dermal, 40% respiratory, 57% ocular, 13% gastrointestinal, 7% neurological and 17% other clinical effects.

### 3.4. Dermal Clinical Effects

The latency period for dermal clinical outcomes varies greatly from immediate/few minutes to 1–2 weeks (Table 3).

**Table 3:** Dermal clinical effects and latency period.

Effect	Expected Latency Period
Blistering rash/bullae [5,13,16,21,29,34,36]	12 h to a week [5,13,16,21,29,34,36]
Erythema/redness [5,16,19,21,22,24,26,28,34,36]	some minutes to 4 days [5,19,21,24,26,28,34,36]
Oedema/swelling [21,24,26,28,34]	1 h to 3 days [21,24,26,28,34]
Burning-sensation [21,22,23,25,33,35,36]	Immediate [21,23,33,35,36]
Burns [5,13,17,19]	>2 days [5,13,19]
Pruritus [21,22,28]	within some days [21,28]
Eczema, seborrhoeic dermatitis [21,36]	4 h to some days [21,36]
Acute generalised exanthematosus pistulitis/skin rash [16,28]	1–2 weeks [16,28]
Allergic contact dermatitis [14,15,21,29,36]	Within a week [14,15,21,29,36]
Dermal irritation/pain [5,16,20,21,26,29]	>24 h [5,20,21,26,29]

Common dermal findings are erythema [5,16,19,21,22,24,26,28,34,36], blistering rash or bullae [5,13,16,21,29,34,36], burning sensation of the skin [21,22,23,25,33,35,36], dermal irritation with or without pain [5,16,20,21,26,29], and burns [5,13,17,19]. Other findings are swelling or oedema [21,24,26,28,34], pruritus [21,22,28], skin rash [16,28] eczema or seborrhoeic dermatitis [21,36], lichenification [28], erythymatous scar [22], allergic contact dermatitis [14,15,21,29,36] acute generalized exathematosus pistulitis (AGEP) [16] and chemical leukoderma [21]. Watson *et al.* reported seven cases which describe subjects exposed at work and facing cutaneous reactions [21]. In this article, three cases of allergic contact dermatitis are described, along with one case of irritant contact dermatitis, leukoderma and seborrhoeic dermatitis respectively. Moreover, there is described a possible increased susceptibility to CS in persons with rosacea. Symptoms with their expected latency period are presented in Table 3. In general a wide variation in the latent period of clinical effects has been found (6 h to 7 days) [5,13,16,21,29,34,36]. There are also cases of acute generalised exanthematosus pistulitis with symptoms beginning 3 weeks after exposure [16].

### 3.5. Respiratory Clinical Effects

The latency period for respiratory clinical effects varies from immediate/few minutes to 2 weeks (Table 4). Clinical findings described from respiratory system are cough [18,23,28,32,35] and dyspnoea or chest tightness [21,27,28,30,32,35]. Other findings include respiratory irritation [20,24,25], reactive airways dysfunction syndrome (RADS) [32,35], hypersensitivity reaction with pneumonitis and bronchocostriction [28], laryngospasm [23] and laryngeal obstruction [18]. Less severe symptoms described are runny nose [21] and sore throat [32] or burning sensation of the throat [24,25,33,35]. It is interesting to state that in one article eight out of 23 young people exposed indirectly needed oxygen therpay [30]. Moreover, in another article there was one subject who faced laryngospasm during removal of tracheal tube after having being exposed to CS [23]. Finally, in the case with the described RADS, the patient continued to face symptoms two years after exposure and needed daily medication [32].

**Table 4:** Respiratory clinical effects and latency period.

Effect	Expected Latency Period
Cough [18,23,28,32,35]	Immediate to 2 days [18,23,28,32,35]
Dyspnoea,chest tightness [21,27,28,30,35]	Immediate/within minutes [21,27,28,30,35]
Respiratory irritation [20,24,25]	Within minutes [24]
Laryngeal obstruction [18]	About 3 weeks [18]
Hypersensitivity reaction with pneumonitis and bronchocostriction/ RADS [28,32,35]	1–2 weeks [28]
Laryngospasm [23]	12 hours after exposure, during anaisthesia [23]
Sore throat/burning of the throat [24,25,32,33,35]	Immediate/within minutes [24,32,33,35]

### 3.6. Ocular Clinical Effects

The latency period for the development of ocular clinical outcomes varies from immediate/few minutes to less than 24 h (Table 5). There are many reports of various ocular symptoms: lacrymation [18,21,23,24,33] (or “runny eyes”), eye irritation [20,24,25,30,33,35] conjunctivitis [16,18,21,24,28,31,33] and stinging of the eyes [21,23,27,33]. There are also reposts of blepharospasm with excessive blinking of the eyes [18,33], keratitis [34] and transient reduction of vision [5]. One more case of vision reduction is described but was caused due to periorbital oedema [26]. Vision returned to normal within two days in both cases (Table 5).

**Table 5:** Ocular clinical effects and latency period.

Effect	Expected Latency Period
Lacrymation [18,21,23,24,27,33]	immediate [18,21,23,24,27,33]
Blinkng/ blepharospasm [18,33]	immediate [18,33]
Sting of the eyes [21,23,33]	Immediate [21,23,33]
Eye irritation [20,24,25,30,33,35]	immediate/within minutes [20,24,30,33,35]
Reduced vision [5]	>24 h [5]
Conjuctivitis [16,18,21,24,28,31,33]	some minutes [18,21]

### 3.7. Gastrointestinal Clinical Effects

There is a case report where gastrointestinal symptoms such as diarrhea, abdominal pain, nausea and vomiting are reported [24]. However, this describes a case of CS ingestion and not an exposure during riots. In this case headache is also reported. Other gastrointestinal symptoms described are abdominal pain [33], loss of appetite [18,28] tender lips and numbness in the tongue [21].

### 3.8. Multisystem Hypersensitivity Reaction

There is a case report on a persistent multisystem hypersensitivity reaction to CS [28]. In this case apart from respiratory hypersensitivity with bronchospasm, toxic chemical hepatitis and diffuse dermatitis (probably because of systematic sensitization with CS) with hypereosinophilia were also reported. The systematic phase of his disease started one week after exposure and lasted for 6 months. During this period the patient was admitted to the intensive care unit.

### 3.9. Long Term and Life Threatening Effects

Interestingly, only 57% of the case reports provided information on long term consequences after exposure to CS (Figure 2). In addition, 70% of the case reports included available information on the need for hospitalization after exposure to CS. Some cases are discharged without hospitalization and are treated as outpatients or require hospitalization for less than 24 h. More severe cases needed better monitoring of their clinical findings and were hospitalized for 5 days to 2 weeks [16,26]. In the case report written by Panreix-Spake *et al.* the average hospitalization period for the 11 patients was 6 days [34]. Three cases, which referred to long-term consequences of riot control agents and specifically CS spray, have been published [28,32,35]. The first by Hill *et al.* describes a multisystem hypersensitivity reaction which lasted for more than 6 months with hospitalization need even three months post-exposure. This case also proved to be a life-threatening one given that the subject needed to enter an intensive care unit [28]. There are two cases of reactive airways dysfunction syndrome presented by Roth *et al.* and Hu and Christani which lasted for more than two and three years, respectively, requiring multiple hospitalisations [32,35]. Allergic contact dermatitis could also be considered as a long-term effect especially when occupational exposure takes place [14,15,21,29,36]. Karaman *et al.* described a case of serious laryngeal and bronchial obstruction which presented 21 days post-exposure and required laryngoscopic examination and bronchoscopy. Moreover this case proved to be a life threatening condition and tracheotomy was needed [18]. It is interesting to note that subjects reported in references [18,28,32,35], and some subjects reported in the work of Watson *et al.* [21] were exposed to CS in enclosed/confined spaces.

### 3.10. Complications during Anaesthesia and Exposure to CS

Bhattacharya and Hayward described the problems experienced with the anaesthetic management of a patient previously exposed to CS gas [33]. In particular, the intubation of this patient was difficult due to the presence of CS in the oropharynx which caused the anaesthetist to suffer severe blepharospasm and lacrimation. In addition, attempts to pass a nasogastric tube, using a laryngoscope failed because of the lacrimation and blepharospasm experienced a by the anaesthetist. Davey and Moppett reported the case of a young man admitted to hospital for treatment [23]. The patient required urgent surgery. He was exposed to CS spray used by police 10 h before induction of anaesthesia. Marked laryngospasm occurred when the tracheal tube was removed by the anaesthetist at the end of the operation and the attending physician experienced lacrimation and burning sensation. These clinical effects on the anaesthetist made the re-intubation of the patient difficult and a senior anaesthetist was called and he removed the tracheal tube and replaced it with a laryngeal airway mask. The physician experienced lacrimation and burning sensation while the patient again developed laryngospasm which resolved after the application of continuous positive airway pressure. Barlow also describes difficulties with anaesthesia because of symptoms experienced by the physician [27].

### 3.11. Descriptive Studies

Zekri *et al.* described 96 patients with acute burn injury occurred resulting from the use of CS in a Hong Kong detention centre of refugees [45]. The average age was 19 years with ages ranging from <1 to 51 years old. Among them two patients younger than 10 years who were admitted to the burns unit. In addition, 46% of the burns were caused by flames from canister explosion, 40% from direct hit and 14% from spray use. Moreover in 8% of the cases the burns were distributed to the face. Anderson *et al.* described clinical effects found in 184 patients after a riot in a Hong Kong refugee detention center [44]. It is believed that the two studies [44,45] are based on the same dataset. A total of 184 patients who reported major symptoms from a total of 1500 subjects (Attack Rate = 12.3%) exposed to CS were interviewed. The most common symptoms described were cough (38%), headache (29%), shortness of breath (21%), chest pain (19%), sore throat (15%), fever (13%), haemoptysis (8%) and haematemesis (4%) and the most common signs were burns (52%) and inflamed throat (27%). In addition, 10 patients (5.5%) had contact dermatitis. Patients were interviewed from eight hours to 19 days after exposure with an average of five days. The maximum duration of symptoms was 22 days for cough, 33 days for shortness of breath and 38 days for sore throat, respectively. It is of note that among the patients was a 3 month old wheezy baby who had confirmed haematemesis. Hankin *et al.* reported on the investigation of an inadvertent secondary exposure to CS among workers in a retail store. The workers became ill after the delivery of imported furniture [39]. Twenty one people were exposed secondarily when handling cargo which presumably had been exposed to CS. In particular, it was assumed that CS had been used by immigration officials in order to detect illegal stowaways in the vehicle transporting the cargo. A questionnaire was sent retrospectively to employees requesting information on condition of exposure and clinical symptoms. In addition, controls, that didn’t report symptoms, were asked about having been in contact with the cargo. Experiencing symptoms was significantly associated with having been in contact. The more prevalent symptoms among exposed employees were eye irritation, itching nose, sneezing, running nose and eyes, itching skin, burning throat, and skin reddening. The mean time between exposure and symptoms onset ranged from 5 to 30 min. In addition, the duration of symptoms ranged from 1 to 4 h. Apart from exposed workers four members of the public were experienced secondary exposure to CS [39]. Thomas and co-workers reported on a cluster of cases during military training of 38 Marines [43]. The training program included exposure to CS dispersed via thermal canisters followed by strenuous exercise. The purpose of the training was to test the ability of the Marines to quickly don their masks and to develop confidence regarding the mask’s effectiveness. Nine out of 38 Marines (Attack Rate = 25%) were hospitalized due to the development of pulmonary syndrome with symptoms of dyspnea, cough and hemoptysis. The marines did not report any symptoms at rest. Clinical signs and symptoms began to appear during and after periods of strenuous exercise performed up to 84 hours after exposure to CS. Five trainees reported hemoptysis, and four Marines developed acute hypoxia and were admitted to the intensive care unit. The five others were admitted to the medical ward for observation. All hospitalized Marines were improved rapidly and were discharged on light duty until their re-evaluation. Their hospitalization length ranged from 24 to 72 h. Wheeler and co-workers reported data from the National Poisons Information Service London (NPISL) [7]. The authors collected data on 597 patients enquires made to NPIS (L) in 1997 related to clinical effects after exposure to crowd control agent including CS which in 1996 started to be used by various English police forces. The descriptive analysis of the data demonstrated that the majority (76%) of the clinical effects occurred within 6 h post exposure while 24% occurred more than 36 hours after exposure. The univariate analysis of the data showed that dermal, and gastrointestinal clinical effects were significantly more prevalent after 6 h of exposure. It is of note that cardiac clinical effects have been recorded both within 6 h of exposure (hypotension, tachycardia) and afterwards (chest pain). These preliminary results led authors to conduct a further study to investigate the clinical effects of exposure to riot control agents and CS. In this study Euripidou *et al.* from London’s National Potions Information Service analyzed characteristics and clinical features of 152 persons during the period January-September 1998. [40]. The mean age of subjects was 26.3 years old and they were predominantly males (77%). Subjects (n = 152) were divided into two subgroups; the first group of persons was sprayed by the police (n = 93; 61%) and the second comprised of persons who faced not police related exposure (n = 59; 39%). The results of the study indicated that subjects who have been sprayed by the police were more likely to develop erythematous dermatitis (OR = 7.57; 95% CI = 2.34–24.51) and blisters (OR = 5.67; 95% CI = 1.2–26.84) in comparison to the subjects not sprayed by the police (reference category). Furthermore, the subgroup of subjects that have been sprayed by the police forces recorded an almost 3 fold risk of adverse outcome in comparison to the reference group (OR = 2.9; 95% CI = 1.41–5.97). Moreover, subjects experienced police spray incidents had an almost 2 fold risk of referral for further treatment to another specialist department (OR = 1.91; 95% CI = 0.96–3.08). Concerns have also been raised about psychiatric effects of CS gas. In a study among claimants in a joint court action against the police force, Nathan *et al.* reported that stress during the exposure, and also post-traumatic stress disorder were documented even three years after exposure to CS [41].

### 3.12. Analytical Studies

We identified only one analytical study that dealt with the clinical effects of exposure to CS gas. In particular, Karagama *et al.* in a prospective cohort study described the short and long term clinical effects among 34 subjects that experienced exposure to CS gas in a bus during altercation with the police [42]. The subjects were divided in two categories according to the type of exposure to CS. Subjects that were hit directly in the face by the spray comprised the direct contact group (n = 10), while the others the indirect contact group (n = 24). Ocular, and respiratory symptoms were prevalent among direct and indirect exposed subjects during the first hour after exposure. The subjects have been followed 1 month and 10 months after exposure. At one month after symptoms were frequent among both groups, however oral symptoms were reported more frequently by the subjects belonging to direct exposure group (50% *vs.* 0%; *p* < 0.001). At ten months after exposure, ocular, respiratory symptoms and other symptoms were reported by nine subjects. Three (Attack Rate = 30%) belonged to the direct exposure group and six (Attack Rate: 25%) to the indirect exposure group. An article by Hout *et al.* describes the incidence of acute respiratory illness (ARI) after CS exposure in US army military training populations [37]. Incidence increased after exposure (*p* < 0.01) and was positively correlated with CS concentration (*p* = 0.03). These results lead to a second article by the same team in which All Army Activities Message (ALARACT) was implemented in order to reduce CS concentration [38]. Concentration was reduced 10-fold and the risk for ARI was significantly reduced if compared with the risk pre-exposure. Post-exposure ARI incidence was increased with statistically significant attributable risk upon concentrations above the Threshold Limit Value Ceiling (TLV-C) (0.39 mg/m^3^). The risk of ARI was positively correlated with CS concentration.

## 4. Discussion

Our systematic review indicates that CS affects mainly the skin, the eyes and the respiratory system. The skin effects of exposure to CS could be of irritant or sensitizing type. It is of note that dermal clinical effects following exposure to CS could affect the work fitness of police employees [21]. In addition there is evidence that secondary exposure to CS could be a notable occupational hazard for health care workers (emergency department personnel, and anaesthetists). Moreover, previous exposure to CS could be associated with post-operative complications among patients that experienced previous exposure to CS. Long-term clinical effects have been found in the literature. For instance, dermal findings such as erythema and rash persisted for almost a month [5]. In addition, dermatitis or pneumonitis could last for six months [28,32,35]. Anderson *et al.* reported a case of cough for more than a month [44]. In all these cases exposure happened in confined or enclosed space. Life threatening situations have been published; these include laryngeal and bronchial obstruction [18], laryngospasm [23], as well as five intensive care unit admissions [43]. In addition there is evidence that subjects sprayed by the police have recorded higher odds of referral for further treatment to another specialist department in comparison to their counterparts that were not sprayed by the police [40]. There is a scarcity of analytical studies in the field of health effects of CS gas. The analytical study by Karagama *et al.* indicated-not statistically significant long-term clinical effects which lasted 10 months post exposure [42]. However it should be noted that the number of subjects in this study was small. CS effects on human health seem to be correlated with concentration and a dose-response pattern has been found [37,38]. In addition, interventions which decreased concentration also decreased the relative risk of post exposure incidence of ARI [38].

Interestingly, results from a project conducted by the Turkish Thoracic Society showed that subjects who were exposed to tear gas including CS reported higher rates of respiratory symptoms and abnormal lung function results in comparison to the control group [46]. Further, study form United Kingdom investigated the effects of incapatitant spray (no differentiation has been made between CS and Pelargonic Acid Vanillyamide—PAVA). The authors concluded that the effects of incapacitant sprays used in the context of law enforcement last longer that generally believed [47]. Last, a recent prospective study among Army recruits from United States indicated that recruits had an almost 2.5 fold increased risk of being diagnosed with Acute Respiratory Illnesses (ARIs) after exposure to CS compared to the period of training preceding exposure [38]. We acknowledge the fact that it is difficult to define a confirmed case in terms of exposure to CS. According to the Center of Disease Control [48] a probable case is a clinically compatible case in which a high index of suspicion exists for riot-control agent exposure, or an epidemiologic link exists between this case and a laboratory-confirmed case. Confirmed is a case in which laboratory tests have confirmed exposure. However, because of the unavailability of any routine specific laboratory test for CS it is difficult to define a confirmed case. Recent studies have attempted to test an analytical method for the measurement of urinary metabolites of CS [49].

The vast majority of the studies in this review were case reports. Case reports represent a low level of epidemiological evidence. Given that the exposed population is not known we cannot calculate attack rates based on case reports. Furthermore, our systematic review revealed that a considerable part of case reports failed to include essential information (e.g., latency period, duration of symptoms). Better surveillance of the subjects exposed to CS and tear gas and conduction of cohort studies among exposed populations (e.g., military/police trainees, police officers, demonstrators, health care workers) are needed in order to properly evaluate the biological effects of exposure to CS among various exposed population subgroups.

## 5. Conclusions

This is the first systematic review in medical literature aiming to evaluate the health hazards of CS which is used for both riot control and military/police training. A significant function of a systematic review is the establishment of further research needs. In this review we assembled and discussed 39 studies. The majority of them were case reports there were few descriptive studies and only one analytical study. It is of note that the analytical study revealed long term clinical effects with Attack Rates ranged from 25%–30%. Also a considerable part of case reports failed to include essential information (e.g., latency period, duration of symptoms). Moreover, long term and life threatening health effects have been recorded. Police officers, demonstrators, bystanders, health care workers and surgical patients could be harmed from exposure to CS. The establishment of surveillance schemes for the registration of the health effects and conditions of exposure among subjects exposed to CS and the completion of cohort studies among exposed populations (e.g., police officers, demonstrators, health care workers) would further illuminate the full health consequences of exposure to CS.
